# Prevalence of *Candida albicans* in High-Risk Human Papillomavirus-Positive Women: A Study in Diyarbakır Province, Turkey

**DOI:** 10.1155/2023/9945561

**Published:** 2023-10-10

**Authors:** Efdal Oktay Gultekin, Tarik Mecit, Behzat Can

**Affiliations:** ^1^Toros University Vocational School of Health Services, Department of Medical Services and Techniques, Mersin, Turkey; ^2^Department of Physiology, Faculty of Medicine, Biruni University, Istanbul, Turkey; ^3^Department of Gyneacological Oncology, Gazi Yaşargil Training and Research Hospital, Diyarbakir, Turkey

## Abstract

The human papillomavirus (HPV) is a significant public health concern due to its association with the development of cervical cancer. Although inflammation caused by *Candida* spp. has been shown to facilitate oncogenesis, the interactions between HPV and *Candida* spp. remain unclear. This study aimed to determine the prevalence and genotype distribution of HR-HPV infection HR-HPV-positive*Candida albicans* in HR-HPV-positive individuals in Diyarbakır province in Turkey. Cervical samples were taken from 350 participants aged 20–69 years who applied to Diyarbakır Gazi Yaşargil Training and Research Hospital, Gynecology and Obstetrics Clinic. For detection of HPV presence and HR-HPV genotyping, PCR/direct cycle sequencing was used. E6/E7 mRNA expression of HPV-16, -18, -31, -33, and -45 was determined by type-specific real-time NASBA assay (NucliSENS EasyQ(®)HPV v1.1). The presence of *Candida albicans* in cervical specimens of HR-HPV-positive women was investigated by RAPD-PCR and culture methods. *Results*. Of the 350 women who participated in the study, 24% were HPV positive and 10.5% were found to be HR-HPV positive. HR-HPV positivity was most frequently detected in the age range of 40–49 years. Among HR-HPV-positive women, *C. albicans* was found in 59.4%. *Conclusion*. The most frequent HR-HPV genotype was HPV16, followed by HPV31. Of women who tested positive for HR-HPV, *C. albicans* was discovered in 59.4%. *C. albicans* infection may occur when the immune system is weakened or the balance of the vaginal flora is disturbed, increasing tissue damage in the vaginal area and the risk of carcinogenesis of HR-HPV. Therefore, knowing the presence of *Candida* infection in HR-HPV-positive women is essential to plan the treatment and prevent the risk of secondary disease.

## 1. Introduction

Cervical cancer (CC) is the second most common malignancy affecting women, following breast cancer. It is characterized by the most significant morbidity and mortality rates among all tumors affecting the female genital tract [1]. The leading cause of cervical cancer has been revealed to be human papillomavirus (HPV) infection, which has led to the recognition of CC as a malignancy with a preventable and treatable etiology. Currently, most countries support the prompt deployment of CC screening, using a thorough strategy that includes cervical cytology and HPV testing [2]. An increasing amount of evidence suggests that HPV vaccinations may significantly reduce the prevalence of HPV infections. Nevertheless, many poor nations have been hesitant to implement widespread vaccination programs [3]. Hence, it is crucial to identify the correlated risk factors of high-risk human papillomavirus (HR-HPV) infection promptly and proactively and effectively treat chronic HR-HPV infection to avoid CC [1].

Human papillomavirus (HPV), widely recognized as the most frequent sexually transmitted infection worldwide, has been found to have detrimental effects on individuals' personal and social lives [3]. Human papillomavirus (HPV) is a member of the Papillomaviridae family, characterized by its small size and double-stranded DNA structure. It can be classified into two distinct groups: low-risk HPVs (LR-HPVs) and high-risk HPVs (HR-HPVs). LR-HPVs are primarily responsible for the development of cutaneous and anogenital warts. At the same time, HR-HPVs are associated with the development of various cancers, including anogenital cancers (such as anal, vulvar, vaginal, and cervical cancers) as well as oropharyngeal cancers [4]. To date, about 200 genotypes of HPV have been identified, with approximately 40 of those infecting the genital tract [2]. Low-risk (LR) HPV types encompass a range of viral strains, namely, types 6, 11, 42, 43, 42, and 44. The HR-HPV types encompass a range of strains, namely, types 16, 18, 31, 33, 34, 35, 39, 45, 51, 52, 56, 58, 59, 66, and 68. Certain kinds of HPV that are less commonly associated with the development of malignancies are classified within the high-risk category. However, they are regularly detected in squamous intraepithelial lesions [3].

*Candida* spp. is one of the sexually transmitted infectious agents and is a part of the human commensal flora, causing superficial and systemic infections. It has been suggested that these microorganisms alter the inflamed cervical epithelium, which increases the chance of HPV infection by enabling the virus to enter the basal cells of the epithelium [2, 3]. Despite these hypothetical possibilities, it is currently unclear how HPV and *Candida* spp. interact to cause cervical cancer [1–5].

This study aimed to determine the prevalence and genotype distribution of HR-HPV infection and *Candida albicans* in HR-HPV patients in Diyarbakır province in Turkey.

## 2. Methods

### 2.1. Study Population

Cervical samples were taken from 350 participants aged 20–69 years who applied to Diyarbakır Gazi Yaşargil Training and Research Hospital, Gynecology and Obstetrics Clinic.

The Ethics Committee of Biruni University's Faculty of Medicine approved this study (approval number: 2023/82-20; approval date: 19/07/2023). Before the cervical specimens were taken, all participants provided their written informed consent after being informed of the study's goals and procedures.

All participants completed a questionnaire asking about their sociodemographic and clinical characteristics, including age, coit age, number of partners, use of condoms, oral contraceptives, intrauterine devices, and smoking history.

### 2.2. Specimen Collection and Processing

The “Thin PrepTM Pap Test” solution, utilized in an FDA-approved, commercial liquid-based cytological procedure, collected cervical specimens from the patients to detect cervical HPV DNA and sexually transmitted illnesses. During the colposcopic examination, the patients were positioned in the lithotomy posture. The sample was obtained by rotating the brush 360° for 10 seconds in the transformation zone of the squamocolumnar junction and ectocervix after entering the endocervical canal with the aid of the inspection speculum. The brush's tip was cut off and thrown into the mixture. Centrifugation separated the Thin PrepTM Pap Test fluid from the cell pellets for 10 min at 4000 g. Before DNA extraction, the samples were divided into two tubes and stored in the refrigerator at −20°C.

### 2.3. HPV Genotype Determination

Direct cycle sequencing was used to determine the HPV genotype in the presence of MY or GP amplicons. Internal PCR primers (MY09 or GP5+) and the BigDye Terminator v3.1 Cycle Sequencing kit (Applied Biosystems, Foster City, CA, USA) with dye terminator dideoxynucleotide were used to analyze PCR products for cycle sequencing in accordance with the manufacturer's directions for sense sequence analysis. An automated capillary sequence reader called the ABI PRISM 3130XL Genetic Analyzer (Applied Biosystems, Foster City, CA, USA) was used to capture the data from cycle sequence reactions. The obtained nucleotide sequences were compared with the HPV reference sequences already present in the GenBank database using the Basic Local Alignment Search Tool (BLAST®) software (https://blast.ncbi.nlm.nih.gov/Blast.cgi). The most comparable and substantial alignment results served as the foundation for identifying the HPV genotype.

### 2.4. High-Risk HPV Detection and Genotyping

The detection of E6/E7 mRNAs of the five most prevalent high-risk HPV genotypes 16, 18, 31, 33, 45, and 58 was performed qualitatively, with a commercial NASBA assay (NucliSENS EasyQ HPV; bioMérieux, France) test according to manufacturer's instructions. For E6/E7 mRNA detection, RNA was extracted from 200 *μ*l sample by using a High Pure Viral RNA Kit (Roche Diagnostics GmbH, Mannheim, Germany). Human U1 small ribonucleoprotein (U1A mRNA) was used as an RNA integrity/adequacy internal control.

Cytobrushes were used to collect cervical samples (the Pap test) and were then stored in medium with 95% ethanol at 4°C until processing. The viral extraction, detection, and typing approach has already been reported [6, 7]. The accuracy of the sample was validated, and the DNA was detected using the GH20/PC04 primers in typical PCR amplification of the beta-globin gene. Three sets of primers were used (GP5+/GP6+, MY09/MY11, and pU1M/2R); they were directed towards the viral genome's L1 and E6/E7 regions for determining the presence of HPV. These primers made detecting coinfections and identifying infections with low viral loads possible, increasing test robustness and sensitivity [8]. Each primer's PCR settings and methods have been previously disclosed [6]. At least one set of generic primers was used to type HR-HPV types 16, 18, 31, 33, 45, and 58 in samples that showed positive PCR results [6]. The primers used are shown in Table 1. All PCR experiments employed positive and negative controls to check for contamination and find unexpected findings (the relevant assay was redone if this occurred). By electrophoresis on a 2% agarose gel, the PCR results were shown.

### 2.5. *Candida* Detection by Culture

To determine the presence of *Candida* spp., samples were collected from 350 female patients who had history of high-risk HPV positive. By injecting 2 ml of physiological saline solution (NaCl) into the vagina and recovering 1 ml of the vaginal fluid after scraping the vaginal walls, the vaginal discharge was collected as a vaginal lavage, and before inoculating on Sabouraud agar, first, the lavage on liquid Sabouraud medium was cultured to increase the number of cells. The cells from the liquid medium were grown on Sabouraud agar the following day and incubated for 48 hours at 37°C. By injecting chromagar (BBL ChromagarTM; Becton, Dickinson, Sparks, USA), colonies formed on sabouraud agar were further differentiated for *Candida* species. Chromagar is a differential culture medium that enables the selective isolation of yeasts while also uniquely identifying the colonies of *Candida albicans*, *Candida glabrata*, *Candida krusei*, and *Candida tropicalis* by producing a distinct colony color after incubation for 24 hours at 37°C. Based on colony color and texture, which are formed by the hydrolysis of chromogenic substrates in the medium, strains are presumptively identified. The species were distinguished using the colors green for *Candida* albicans, pink for *Candida glabrata*, pale pink for *Candida krusei*, and blue for *Candida tropicalis*.

### 2.6. *Candida* spp. Detection with PCR

#### 2.6.1. DNA Extraction

The genomic isolation kit provided by Geneaid Company/Taiwan (Cultured Cell Protocol Procedure Ver. 11.21.13) was used to produce and purify the DNA. The commercial kit was used with liquid nitrogen (170 C), as the extremely low temperature helped to avoid DNase activation [9]. According to Sambrook and Russell [10], 2 *μ*l of each DNA sample was used to evaluate the concentration and purity of the DNA using the Nanodrop device (BioDrop/UK). A good yield of the necessary PCR products required a DNA concentration between 50 and 200 ng/l, according to Saiki [11]. As a result, the DNA concentration was decreased to 50 ng/l.

#### 2.6.2. *Candida* spp. PCR

The specific primers and their sequences are chosen in accordance with Table 2. The amplification reaction protocols for particular polymerase chain reaction (PCR) were utilized: initial denaturation: 95°C for 5 min (denaturation: 95°C for 1 min, annealing: 58°C for 45 s, elongation: 72°C for 1 min) (40 cycles, final elongation: 72°C for 10 min), while RAPD-PCR was initial denaturation: 94°C for 5 min, denaturation: 94°C for 1 min, annealing: 36°C for 2 min, and elongation: 72°C for 1 min. 40 cycles; final elongation: 72°C for 10 min.

### 2.7. Statistical Analysis

Clinical information was logged, including age, education level, menarche age, method of contraception, and smoking status. Using SPSS for Windows version 10.0, chi-square and Spearman correlation analysis were performed to analyze the data.

## 3. Result

The average age of the 350 women involved in the study was 45 and varied from 20 to 67. Contraceptive techniques were used as follows: condoms were reported as the preferred method by 46.8% of respondents, while oral contraceptive was chosen by 34% of participants. Intrauterine devices were utilized by 12.8% of individuals. 100 out of 350 women, accounting for 28.5%, were identified as multiparous. There was no statistically significant difference observed between patients who tested positive for HPV concerning variables such as age at menarche, level of education, menopause, contraceptive method, and smoking status. The results of the study indicated a statistically significant relationship (*p* < 0.05) between HR-HPV positivity and having women with multiple partners. HR-HPV-positive women all reported multipartnership. Of 350 women, 84 (24%) were HPV positive and 37 (10.5%) were found to be HR-HPV positive. A statistically significant relationship was found between HPV positivity and age range, and 46 patients were between the ages of 40 and 49 (*p* < 0.05). No statistically significant relationship was found between the use of OCS, condoms, IUD, and HPV positivity (*p* > 0.05) (Table 3).

37 out of 84 HPV-positive patients (44%) had positive HR-HPV results. There were six distinct types identified, with type 16 being the most common (70%); the most common HR-HPV genotype was HPV16 (70%), followed by HPV31 (21.6%), HPV18 (2.8%), HPV33 (2.8%), and HPV45 (2.8%). HPV 58 was not detected in HR-HPV-positive samples.  Table 4 displays the frequencies of various HPV types. Among the HR-HPV types, only HPV-16 positivity was found to have a statistically significant relationship between the age range, and 19 of the patients were in the 40–49 age range (*p* < 0.05) (Table 5).

*Candida* spp. was detected in 25 (67.5%) of the HR-HPV-positive individuals, and 22 (59.4%) were found to be *C*. *albicans*. *Candida albicans* was detected in 19 (73%) of 26 HPV-16 type positive women, which was statistically significant (*p* < 0.05) (Table 6).

## 4. Discussion

About 200 HPV genotypes have so far been identified. Some of these, known as HR-HPVs, have increased carcinogenic potential and infect the vaginal tract. Given the link between HR-HPV and cervical cancer, it will be essential to identify patients with the infection to provide vital information for their prevention and treatment. In this study, samples from 350 patients were tested for 6 HR-HPV genotypes, and 10.5% of the samples returned positive results.

This study did not find a statistically significant relationship between menopause, condom use, oral contraceptives, and IUD use among the sociodemographic factors examined with HPV. This finding contrasts with a previous study conducted by Coser et al. [12], which reported a statistically significant association between HPV and being single or earning an income below a minimum wage. Regarding the behavioral factors, a study conducted by Coser et al. [12] revealed that the age at which individuals engage in their first sexual intercourse and the number of sexual partners they have are not significantly linked to HPV infection. This conclusion aligns with our research, as we found no statistically significant correlation between these parameters. Nevertheless, Silva et al. [13] conducted a study that revealed that the presence of a solitary sexual partner was identified as a mitigating factor associated with a reduced likelihood of developing a high-grade lesion resulting from human papillomavirus (HPV) infection. Shaw et al. [14] conducted a study that revealed a statistically significant correlation between condom usage and the presence of HPV, in contrast to the findings of this study and Coser et al. [12]. According to a survey conducted by Manhart and Koutsky [15] in 2002, it was found that although condom usage protects against genital warts, it does not effectively prevent HPV infection.

Several studies have indicated that using the intrauterine device (IUD) is associated with a protective effect against HPV infection. This phenomenon can be attributed to the robust immunological response elicited by the IUD within the uterine cervix, leading to the prompt eradication of diseases. According to Ortiz and Croxatto [16], alternative research has indicated that persistent infections could potentially facilitate the progression of high-risk premalignant lesions associated with HPV [17]. According to Silva et al. [13], hormonal contraceptives were a protective factor for advancing uterine lesions caused by HPV. According to a study by Bahmanyar et al. [18], evidence suggests a correlation between long-term usage of hormonal contraceptives and heightened susceptibility to HPV infection. The association in question can be elucidated by examining the biological mechanism involving the upregulation of estradiol hydroxylation in cervical cells infected with oncogenic strains of HPV. This, in turn, leads to an enhanced transcription of oncogenic agents.

In this investigation, no significant correlation was found between the patients' assessed personal habits and the presence of HPV. Coser et al. [12] evaluated the relationship between smoking and HPV, and their findings indicated a lack of a link between the two variables. Silva et al. [13] established a correlation between smoking and increased susceptibility to HPV infection.

The findings about the variables obtained at the initial stage of the study were primarily congruent with existing literature on factors that predict the occurrence of any HPV infection, as well as the presence of oncogenic HPV types. Specifically, our results demonstrated relationships between these variables and age and the number of sexual partners [19, 20]. A positive correlation exists between the number of sexual partners and the likelihood of acquiring HR-HPV infections (*p* < 0.05). More information about women's sexual behavior was hindered by the taboo around sexual disclosure. In the current study, the number of single women who participated was low since the annual gynecologic exam as a routine checkup for single women is not a common practice in Diyarbakır province in Turkey. The prevalence of HPV in our study sample was 24%, which was higher than in other studies [21]. HPV rates were found to be highest in the ages from 40 to 49 (54.7%) and were found to be compatible with other studies conducted in Turkey [22, 23]. In many studies, contrary to our data, it has been reported that HPV positivity peaks between the ages of 20 and 29 [23, 24].

More than 40 closely related but genetically distinct HPVs that infect the genital tract have been classified as HR or LR according to their oncogenic potential [25, 26]. HR-HPV was detected in 10% of participants. In our study, the most frequently seen HPV genotypes among women were HR-HPVs. In this study, the most common HR-HPV genotype was HPV16 (70%), followed by HPV31 (21.6%). The data we obtained were compatible with other studies carried out in Turkey [2, 22, 27]. If the immune system fails to clear the HR-HPV types, infections will be associated with a high risk for cervical disease progression leading to cervical cancer [28]. In studies conducted in China and Turkey, the most common HR-HPV types were reported as HPV52 and HPV51, respectively [29, 30].

The prevalence of HPV16 and HPV31 genotypes among European, Mexican, Turkish, and Chinese Uyghur women exhibiting cervical cytologic abnormalities aligns with the results reported in previous research investigations. According to the findings of these investigations, HPV31 showed the second highest frequency [29, 31–37].

Vulvovaginal candidiasis is a prevalent infection that impacts around 70–75% of women within the reproductive age range. Based on estimates, a significant proportion, ranging from 40% to 50%, of the total cases is expected to exhibit recurrences, whereas approximately 5% to 15% are anticipated to have recurring occurrences [38]. The etiological agent responsible for these infections is *Candida* spp., which is classified as a commensal yeast that is a constituent of the indigenous human microbiota. However, under specific conditions, it can transit into a pathogenic state [39]. One instance illustrating this scenario is when an elevated production of female hormones, specifically progesterone, augments the glycogen content within the vaginal mucosa. Consequently, this surplus of glycogen serves as an energy source, thereby facilitating the growth and germination of yeasts. In addition, this phenomenon enhances the adhesion capability of these fungi [40, 41]. The present investigation found that the incidence of *C. albicans* in HR-HPV-positive patients was 59.5%, a value that falls below the global prevalence (65.3%) and the majority in Europe (67.9%) [42]. *Candida tropicalis* (5.4%) and *Candida glabrata* (2.7%) were found to be isolated at very low frequencies, which aligns with the findings reported in the global literature [42]. The study participants frequently reported experiencing symptoms such as the presence of lumpy discharge and itching in the vulvar region. When evaluating the clinical manifestations observed in these individuals, there was a lack of differentiation between *Candida* spp. infection and bacterial infection. Recurrent inflammations have been found to promote cell proliferation, enable the growth of malignant cells, and trigger the release of cytokines, chemokines, and free radicals. These findings suggest that individuals with abnormal microbiota may have more incredible cellular changes than those with normal microbiota [43]. An illustration of this phenomenon can be observed in instances of recurrent vulvovaginal candidiasis, which can be attributed to the elevated pathogenicity exhibited by strains of *C*. *albicans*. These strains can penetrate mucosal barriers, induce edema, cause exfoliation of cells [17], and promote heightened production of virulence-related factors, including proteases, phospholipases, and the formation of biofilms. In this study, the presence of *Candida* spp. was investigated by performing both *Candida* PCR and *Candida* culture in vaginal discharge in patients with HR-HPV for cervical cancer. The findings revealed that the prevalence of positive cases was 59.5% for *C*. *albicans*, 2.7% for *Candida glabrata*, and 5.4% for *Candida tropicalis*. These percentages were slightly higher compared to those typically observed in patients without HPV, as reported by Melo et al. [44].

The research conducted by Nogueres et al. [45] establishes a correlation between the characteristics of women affected by inflammatory conditions caused by *Candida* spp. and their medical history, mainly about sexually transmitted diseases. Notably, infection by the HPV emerges as a prominent factor. Voog et al. [46] postulated the potential of *Candida* spp. infection to trigger the reactivation of latent HPV infections. Previous research has identified these infections as cofactors in the development of cervical neoplasias about HPV, as noted by Sobel [40] and Melo et al. [44] In our study, *Candida albicans* was found in 73% of HPV-16-positive patients. The presence of noteworthy correlations between bacterial vaginosis and HPV 58, as well as between *Candida* spp. and HPV-16 and HPV-53, underscores the necessity of incorporating sexually transmitted disease management into a comprehensive cervical cancer prevention program [47]. *Candida* spp. infection is relatively frequent and commonly observed in asymptomatic colonization and vulvovaginal candidiasis. However, it has been observed that specific species of *Candida* spp. exhibit a higher level of pathogenicity than other species. These particular species can induce the production of hyphae and pseudohyphae and enhance proteolytic activity and antigen modulation [48]. The characteristics above facilitate the ability of *Candida* spp. to infiltrate the mucosal surface and initiate mucosal inflammation, characterized by swelling, redness, and shedding of cells. Several authors, notably Engberts et al. [5], have examined the involvement of *Candida* spp. in the pathogenesis of HR-HPV. Their findings indicate the presence of *Candida* spp. It does not correlate with a heightened risk of acquiring HR-HPV.

The study's limitations are that our analysis was performed only on the patients who attended gynecology polyclinics at the health institution in the Diyarbakir province of Turkey. Although our results do not reflect Turkey in general, it is an advantageous study for directing vaccination studies in our region. Our subsequent work will evaluate the types by classifying the samples as pathological. The other limitation of our study is its case-control design. Establishing causality between sexually transmitted infection coexistent with HPV and cervical neoplasia is difficult in a cross-sectional study. However, doing a prospective study to assess the linkage between HPV, other genital infections, and developing high-grade cervical neoplasia will be both time- and resource-intensive.

*Candida* infection is a fungal infection that can occur when the immune system is weakened or the vaginal flora is out of balance. *Candida* infection can increase tissue damage in the vaginal area and increase the risk of cancerization of HR-HPV. The presence of *Candida* infection in an HR-HPV-positive woman may affect treatment planning. *Candida* infection may need to be controlled first to improve the efficacy of treatment. It is also important to monitor HR-HPV infection during treatment of *Candida* infection.

## Figures and Tables

**Table 1:** HPV primers used in this study.

Types	Primer	Primer sequence (5′–3′)	Size (bp)	Region
GP5+	Pr1	TTTGTTACTGTGGTAGATACTAC	150	L1
GP6+	Pr2	GAAAAATAAACTGTAAATCATATTC		

MY09	Pr1	CGTCCMARRGGAWACTGATC	450	L1
MY11	Pr2	GCMCAGGGWCATAAYAATGG		

HPV-16	Pr1	TCAAAAGCCACTGTGTCCTGA	120	E6
	Pr2	CGTGTTCTTGATGATCTGCAA		

HPV-18	Pr1	CGACAGGAACGACTCCAACGA	202	E6-E7
	Pr2	GCTGGTAAATGTTGATGATTAACT		

HPV-31	Pr1	CTACAGTAAGCATTGTGCTAT	155	E5
	Pr2	ACGTAATGGAGAGGTTGCAATAACCC		

HPV-33	Pr1	AACGCCATGAGAGGACACAAG	212	E7
	Pr2	ACACATAAACGAACTGTGGTG		

HPV-45	Pr1	ACGGCAAGAAAGACTTCGCA	134	E6-E7
	Pr2	CACAACAGGTCAACAGGATC		

HPV-58	Pr1	CGAGGATGAAATAGGCTTGG	109	E7
	Pr2	ACACAAACGAACCGTGGTGC		

**Table 2:** *Candida albicans*-specific primers.

*Candida* species	Primer's name	Sequence (5–3′)	Amplified fragment size (bp.)
*Candida albicans*	CABF59	TTGAACATCTCCAGTTTCAAAGGT	515
	CABR110	GTTGGCGTTGGCAATAGCTCTG	

**Table 3:** Analysis of sociodemographic and risk factors of study participants.

Overall	Total *N*: 350 (%)	HPV (+) *n*: 84 (%)	HR-HPV *n*: 37 (%)	*P* value
*Age (years)*				
20–29	27 (7.7%)	8 (9.6%)	4 (10.8%)	*p* < 0.05
30–39	95 (27.1%)	27 (32.2%)	8 (21.6%)	
40–49	79 (22.6%)	46 (54.8%)	24 (64.8%)	
50–59	120 (34.3%)	2 (2.3%)	0 (0)	
60–69	29 (8.3%)	1 (1.1%)	1 (2.8%)	
*Education level*				
Illiterate	47 (13.4%)	9 (10.7%)	2 (5.5%)	*p* > 0.05
Elementary school	126 (36%)	27 (32.1%)	12 (32.4%)	
Secondary school	84 (24%)	24 (28.6%)	11 (29.7%)	
University or higher education	93 (26.6%)	24 (28.6%)	12 (32.4%)	
*Age of sexual debut*⁣^*∗*^				
<20 years	168 (48%)	38 (45.3%)	16 (43.3)	*p* > 0.05
≥20 years	182 (52%)	46 (54.7%)	21 (56.7)	
*Menopause*				
Yes	57 (16.3%)	9 (10.8%)	4 (10.8)	*p* > 0.05
No	293 (83.7%)	75 (89.2%)	33 (89.2%)	
*Smoking habit*⁣^*∗*^				
Yes	53 (15.1%)	17 (20.2%)	6 (16.2%)	*p* > 0.05
No	297 (84.9%)	67 (79.8%)	31 (83.8%)	
*Condom use*				
Yes	164 (46.8%)	40 (47.6%)	15 (40.5%)	*p* > 0.05
No	186 (53.2%)	44 (52.4%)	22 (59.5%)	
*ITU use*				
Yes	45 (12.8%)	10 (11.9%)	4 (10.8%)	*p* > 0.05
No	305 (87.2%)	74 (88.1%)	33 (89.2%)	
*Oral contraceptive use*				
Yes	119 (34%)	28 (33.3%)	7 (18.9%)	*p* > 0.05
No	231 (66%)	56 (66.7%)	30 (81.1%)	
*More than one partner in life*				
Yes	100 (28.5%)	47 (55.9%)	37 (100%)	*p* < 0.05
No	250 (71.5%)	37 (44.1%)	0	

**Table 4:** Distribution of the HPV types in the study population.

HR-HPV types	Frequency (*n*)
HPV-16	26 (70%)
HPV-18	1 (2.8%)
HPV-31	8 (21.6%)
HPV-33	1 (2.8%)
HPV-45	1 (2.8%)
HPV-58	0

**Table 5:** Distribution of HR-HPV types according to age groups.

Ages	Type 16 patients *n*: 26	Type 18 patients *n*: 1	Type 31 patients *n*: 8	Type 33 patients *n*: 1	Type 45 patients *n*: 1	Type 58 patients *n*: 0	*P*
20–29	2	0	1	1	0	0	<0.05
30–39	5	1	2	0	0	0	>0.05
40–49	18	0	5	0	1	0	>0.05
50–59	0	0	0	0	0	0	>0.05
60–69	1	0	0	0	0	0	>0.05
Total	26	1	8	1	1	0	

**Table 6:** Frequency of *Candida* spp. in high-risk HPV-positive women.

	HR-HPV (positive)	HPV-16 *n*: 26 (%)	HPV-18 *n*: 1	HPV-31 *n*: 8	HPV-33 *n*: 1	HPV-45 *n*: 1	HPV-58 *n*: 0	Total
*Candida*	*n*: 25 (67.5%)	*n*: 20 (80%)	*n*: 0	*n*: 3 (12%)	*n*: 1 (4%)	*n*: 1 (4%)	*n*: 0	*n*: 25 (100%)
*Candida albicans*		19 (76%)		3 (12%)				22 (59.5%)
*Candida glabrata*						1 (4%)		1 (4%)
*Candida tropicalis*		1 (4%)			1 (4%)			2 (8%)

## Data Availability

The data that support the findings of this study are available from the corresponding author upon reasonable request.

## Abbreviations

HPV: Human papilloma virus
HR-HPV: High-risk human papilloma virus
PCR: Polymerase chain reaction
CC: Cervical cancer.
