# Corrigendum to “Prevalence of *Candida albicans* in High-Risk Human Papillomavirus-Positive Women: A Study in Diyarbakır Province, Turkey”

**DOI:** 10.1155/cjid/9801739

**Published:** 2025-07-11

E. Oktay Gultekin, T. Mecit, and B. Can, “Prevalence of *Candida albicans* in High‐Risk Human Papillomavirus‐Positive Women: A Study in Diyarbakır Province, Turkey,” *Canadian Journal of Infectious Diseases and Medical Microbiology* 2023, no. 1 (2023), https://doi.org/10.1155/2023/9945561.

The author list incorrectly omitted the author “Tarık Mecit”.

The corrected author list should read: Efdal Oktay Gultekin, Tarık Mecit, and Behzat Can.

The author contributions statement should read as follows:

E. Oktay Gultekin planned the study, supplied the materials, carried out the laboratory work, performed statistical analysis, and wrote the article.

T. Mecit: Project administration, visualization, and writing–review & editing.

B. Can collected the sample, collected the participant data, analyzed the data, collected and processed the data, and critically reviewed the article.

We apologize for this error. The author list and contributions statement in the original article has since been updated accordingly.

